# Neurog1, Neurod1, and Atoh1 are essential for spiral ganglia, cochlear nuclei, and cochlear hair cell development

**DOI:** 10.12703/r/10-47

**Published:** 2021-05-11

**Authors:** Karen L Elliott, Gabriela Pavlinkova, Victor V Chizhikov, Ebenezer N Yamoah, Bernd Fritzsch

**Affiliations:** 1 Department of Biology, University of Iowa, Iowa City, IA, USA; 2 Institute of Biotechnology of the Czech Academy of Sciences, Vestec, Czechia; 3 Department of Anatomy and Neurobiology, The University of Tennessee Health Science Center, Memphis, TN 38163, USA; 4 Department of Physiology and Cell Biology, University of Nevada, Reno, NV, USA

**Keywords:** bHLH genes, cochlea development, neuronal differentiation, cochlear nuclei projections

## Abstract

We review the molecular basis of three related basic helix–loop–helix (bHLH) genes (*Neurog1*, *Neurod1*, and *Atoh1*) and upstream regulators *Eya1/Six1*, *Sox2*, *Pax2*, *Gata3*, *Fgfr2b*, *Foxg1*,** and *Lmx1a/b* during the development of spiral ganglia, cochlear nuclei, and cochlear hair cells. Neuronal development requires early expression of *Neurog1*, followed by its downstream target *Neurod1*, which downregulates *Atoh1* expression. In contrast, hair cells and cochlear nuclei critically depend on *Atoh1* and require *Neurod1* and *Neurog1* expression for various aspects of development. Several experiments show a partial uncoupling of *Atoh1/Neurod1* (spiral ganglia and cochlea) and *Atoh1/Neurog1/Neurod1* (cochlear nuclei). In this review, we integrate the cellular and molecular mechanisms that regulate the development of auditory system and provide novel insights into the restoration of hearing loss, beyond the limited generation of lost sensory neurons and hair cells.

## Introduction

Without a doubt, loss of hair cells, in combination with deprivation of sensory neurons and cochlear nuclei, results in severe aging-related hearing loss^1–5^. Various approaches to hearing restoration focus mostly on hair cell regeneration, often without a full appreciation of the apparent interaction of hair cells with sensory neurons and cochlear nuclei^6–8^. For instance, the loss of hair cells also reduces most, but not all, spiral ganglion neurons^9–11^. Furthermore, early loss of sensory neurons massively affects the cochlear nuclei^12^. Thus, the best way of approaching the development/regeneration of hair cells, sensory neurons, and cochlear nuclei neurons is to resolve their dependence on each other: how are the development of hair cells, sensory neurons, and cochlear nuclei related^13–18^?

Three basic helix–loop–helix (bHLH) genes were shown to be crucial for hair cell, sensory neuron, and cochlear nucleus development:

1. *Neurog1* plays a crucial role in sensory neuron development, affects hair cells^19,20^, and has a limited impact on cochlear nuclei^21^.

2. *Neurod1* plays a role in neuronal differentiation, cochlear nucleus development, and hair cell development^16,22,23^.

3. *Atoh1* is essential for cochlear hair cells and cochlear nuclei development^24–26^ and has a limited effect on sensory neurons^27,28^.

**Figure 1.**
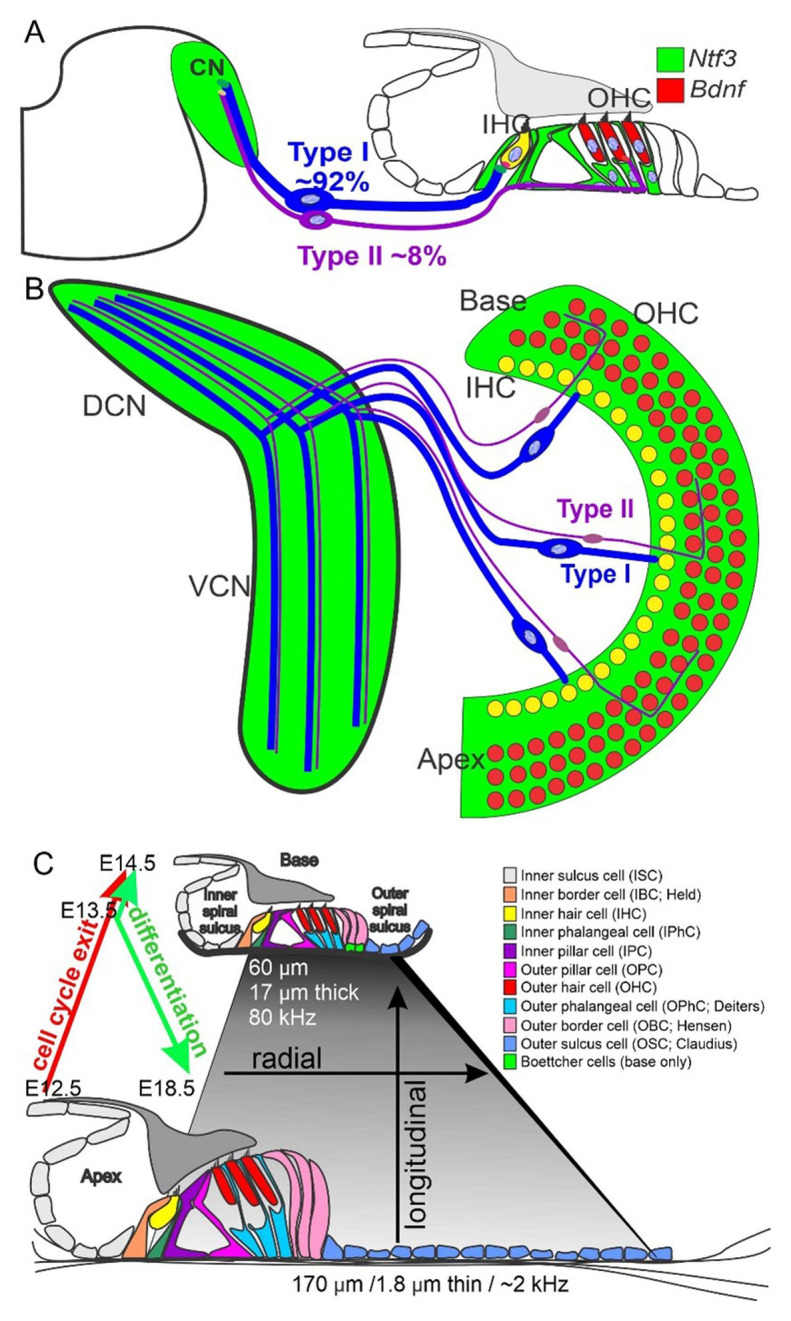
The auditory system revealed in development. Organization of the cochlear hair cells, the spiral ganglia, and the innervation of the cochlear nuclei (**A**). Details show the differential innervation of spiral ganglion neurons to the inner hair cells (IHCs) (yellow, expresses both Ntf3 and brain-derived neurotrophic factor (BDNF)) and outer hair cells (OHCs) (red, expresses BDNF). Note that only Ntf3 (green) is expressed in cochlear nucleus neurons (**B**). After the apex-to-base cell cycle exit (E12.5–14.5), a base-to-apex differentiation of hair cells by *Atoh1* follows (E14.5–18.5) (**C**). In addition, differences in hair cells and supporting cells and the size and thickness of the organ of Corti are depicted (**C**). DCN, dorsal cochlear nucleus; E, embryonic day; VCN, ventral cochlear nucleus. This figure was adapted with permission from Booth KT *et al*.^64^ under the terms of the Creative Commons 4.0 Attribution License (CC BY 4.0) (**A** and **C**) and from Rubel and Fritzsch^12^ (**B**).

Sensory neurons exit the cell cycle from the base to the apex between embryonic day 10 (E10) and E12 in mice, followed by cochlear hair cells from the apex to base between E12 and E14^29^. In parallel, cochlear nuclei exit the cell cycle between E10 and E14^30^. Spiral ganglion neurons project to cochlear hair cells (from base to apex; E13–E16; Figure 1) and nearly simultaneously send central processes to cochlear nuclei (from base to apex; E12– E16)^31–36^. Neurons and hair cells have been suggested to have a clonal relationship because of similarities in bHLH gene expression. This relationship may play a role in neuronal pathfinding for at least the periphery^37^; however, central targeting is less understood but may involve *Neurod1*^16^.

Spiral ganglion neurons depend upon *Neurog1*^19^ and *Neurod1*^22^. In contrast to *Neurog1* null mice^19^, which showed a complete loss of neurons, *Neurod1* null mice^23^ showed residual spiral ganglion neurons extending centrally to smaller cochlear nuclei^16,22^. Unlike *Neurog1*, which is possibly transiently expressed in cochlear nuclei, *Neurod1* was found massively expressed, overlapping with *Atoh1*^26^, *Ptf1*^38,39^, and *Lmx1a/b*^14,25^. Peripherally, it was established that cochlear hair cells critically depend on *Atoh1* (*Math1*)^24^. Furthermore, the length of the cochlea depends on *Neurog1*^19^ and *Neurod1*^22,23^. *Neurog1* is upstream of *Neurod1*^20^, and both are upstream of *Atoh1*^28,40^. *Neurog1* and *Neurod1* truncate *Atoh1* expression^19,27^. Similarly, in the cerebellum, *Neurod1* negatively regulates *Atoh1*^41^, suggesting that these genes interact in many areas of neuronal development. Also, a loss or reduction of cochlear hair cells occurs following the absence of *Gata3*^42^, *Pax2*^43^, *Eya1/Six1*^44^, *Foxg1*^45,46^, and *Lmx1a*^47–49^, and many of these genes and others also affect the sensory neurons innervating them^31,42,43,50–53^.

We will provide a comprehensive review of the interplay of the three bHLH genes (*Neurog1*, *Neurod1*, and *Atoh1*) in the context of spiral ganglia, cochlear nuclei, and cochlear hair cells development. In addition, we will examine the role of other transcription factors (*Eya1/Six1*, *Sox2*, *Pax2*, *Gata3*, *Foxg1*, and *Lmx1a/b*) known to be involved in their development.

## Spiral Ganglion Neurons

### Crosstalk of *Neurog1*, *Neurod1*, and *Atoh1* determines inner ear sensory neuron fate

Both *Neurog1* and *Neurod1* play important roles in sensory neuron development and differentiation. All inner ear sensory neurons were lost in *Neurog1* null mice^19^. Similarly, many sensory neurons were lost in *Neurod1* null mice; however, not all neurons were lost^54^. More recent work in *Neurod1* null mice showed that of those neurons that survived, there was an intermingled vestibular and auditory sensory neuron projection to cochlear hair cells^16,27^ and showed a reduced and aberrant central projection to cochlear nuclei^10,16^.

**Figure 2.**
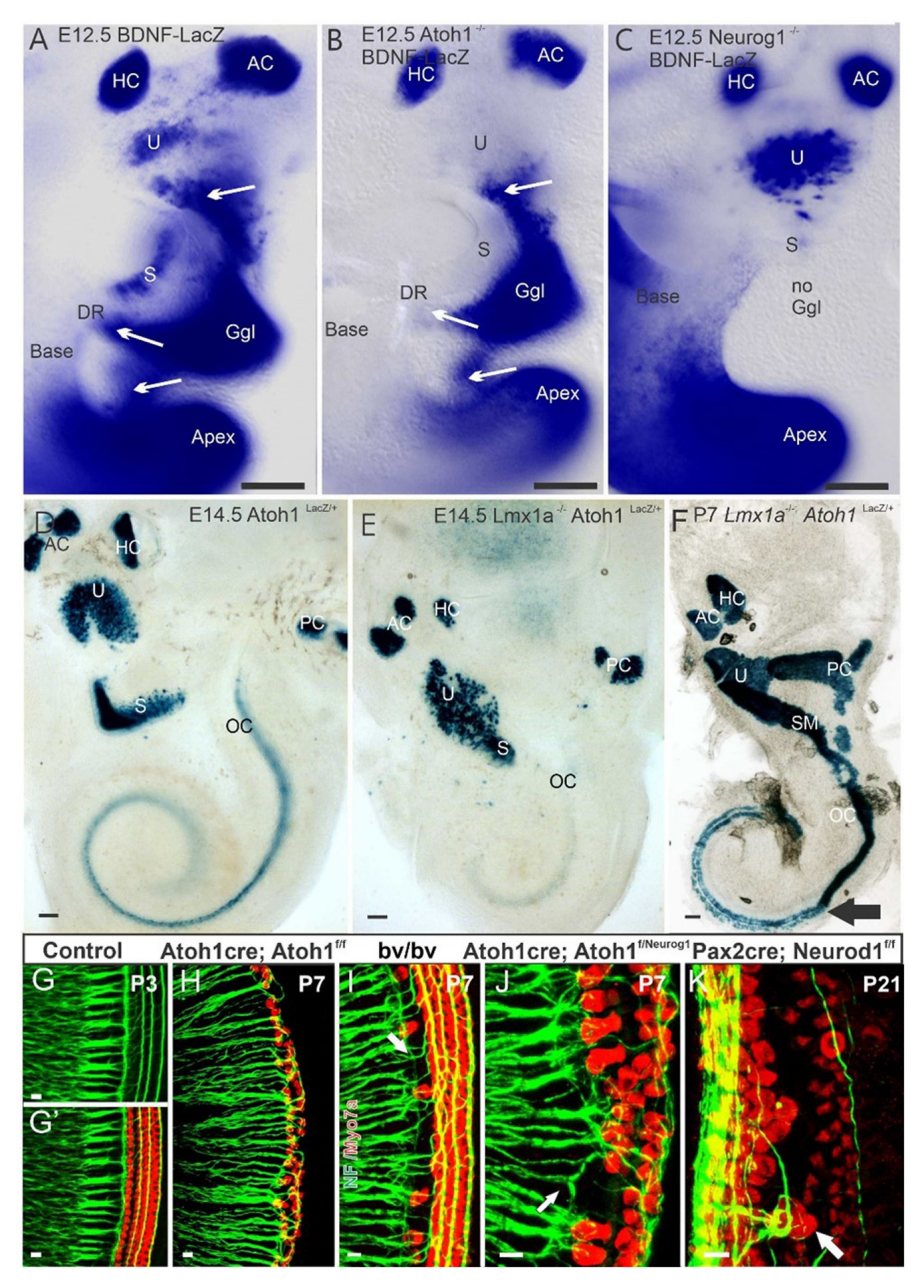
Spiral ganglion neurons depend primarily on *Neurog1* for the development. *BDNF-LacZ* of control mice (**A**) is compared with *Atoh1^f/f^; BDNF^LacZ^* (**B**) and *Neurog1^f/f^; BDNF^LacZ^* (**C**). There is an absence of some, but not all, hair cells in *Atoh1* null mice (**A**, **B**) and loss of sensory neurons and gain and loss of different hair cells in *Neurog1* null mice (**C**). *Atoh1^LacZ^* at embryonic day 14.5 (E14.5) shows near-complete hair cell development near the apex (**D**). In E14.5 *Lmx1a^−/−^* mutants, there is a delayed expression of *Atoh1^LacZ^* (**E**). By postnatal day 7 (P7), the hair cells develop, but there is a fusion of the organ of Corti (OC) with the saccule (SM) (**F**). Detailed comparisons show normal inner ear afferents in controls (**G, G′**), reduced afferents in *Atoh1-cre; Atoh1^f/f^* “self-termination” (**H**), an expansion of afferents to outer hair cells in the absence of inner hair cells in Bronx waltzer (*bv/bv*) (**I**) and *Atoh1-cre; Atoh1^f/kiNeurog1^* (**J**) mutants and altered innervation and cell type formation in *Neurod1* conditional deletions (**K) (arrows**). AC, anterior canal crista; DR, ductus reuniens; Ggl, ganglion; HC, horizontal canal crista; P, postnatal day; PC, posterior canal crista; S, saccule; U, utricle. This figure was adapted with permission from Jahan *et al.*^10^ (**A**–**C**), from Matei *et al*.^28^ (**D**–**F**), and from Copyright Clearance Center: Springer Nature, Cell and Tissue Research, Nichols *et al*.^49^, Copyright © 2008, Springer-Verlag (**G**–**K**).

What is unknown is whether there is a *direct* role of *Atoh1* in sensory neuron development or whether it is indirect. Hair cells depend on neuronal innervation for long-term maintenance^55–57^. Similarly, neurons depend on hair cells and supporting cells for their maintenance^12^. Logically, one would assume that the absence of hair cells will eventually cause degeneration of many neurons because of a lack of neurotrophic support. *Atoh1* null mouse embryos, which lack hair cells, showed reduced *Bdnf-lacZ* staining and reduced hair cell innervation in the basal turn of the cochlea (Figure 2). The apex, which retained *Bdnf-lacZ* staining in undifferentiated cells of these mice, showed a denser spacing of spiral ganglion neurons, suggesting that *Bdnf* expression may not depend on *Atoh1* in the apex^58^. Conditional deletion of *Atoh1* resulted in residual innervation correlated to residual hair cell formation^11,27^, demonstrating that near-normal residual cochlear hair cells receive innervation from a surprisingly large number of neurons^27^. *Pou4f3* (*Brn3c*) null mice, which develop only immature hair cells and have limited expression of neurotrophins^59^, show little effect on innervation patterns beyond the lack of innervation to outer hair cells (OHCs) birth. The absence of inner hair cells (IHCs), through the loss of *Atoh1* or in Bronx-waltzer mutants, results in spiral ganglion projections to OHCs and disorganized central projections^10,60,61^ (Figure 2). Interestingly, replacing an allele of *Atoh1* with *Neurog1* in *Atoh1^kiNeurog1^* mice showed a different pattern of spiral ganglia projections to reach out the organ of Corti^62,63^ (Figure 2), consistent with a reduction in the number of neurons and hair cells^16^.

Furthermore, although *Atoh1/Neurod1* double null mice have no differentiated hair cells, they retain cochlear nuclei and a diminished spiral ganglion with aberrant innervation^27^, suggesting an uncoupling of innervation and hair cell differentiation. The inactivation of both bHLH transcription factors in double *Atoh1/Neurod1* null mutants uncouples fiber growth and expansion of remaining neurons^27^ that could be useful for hair cell restoration^3,5,65,66^. More recent data using *Rosa^CreER^; Rainbow* mice showed clones of spiral ganglion neurons and hair cells in the organ of Corti, suggesting that they arose from a typical progenitor cell^67^. Initially, the meaning of the transient expression of apparently cochlear-derived neurons was unclear.

In contrast to the loss of spiral ganglion neurons in mice lacking *Neurog1*^19,28^, overexpression of *Neurog1* in immortalized multipotent otic progenitors (a cellular system for spiral ganglion neuron differentiation) drives proliferation via increased *Cdk2*. It promotes neuronal differentiation through the expression of *Neurod1*^68^. These findings suggest that *Neurog1* can promote proliferation or neuronal differentiation and possibly impact hair cells without affecting cochlear nuclei^68,69^. It appears that a set of data support the transformation of astrocytes into neurons in *Neurod1*^70^ and *Neurog2*^71^. The induction of neuronal proliferation and otic progenitor cell transplantation is a potential strategy to replace lost spiral ganglion neurons.

Recent work on the characterization of neuronal and hair cell progenitors revealed insights into early gene expression during neuronal development^7,72^. Markers for spiral ganglion neurons, *Isl1*^73,74^ and *Gata3*^9,75,76^, were detected in developing neurons, although *Neurod1* was seen in only the youngest neurons**^7^.

In summary, the known deletion of spiral ganglion neurons in *Neurod1* and *Neurog1* null mice^27,28^ suggests these as potential genes for the induction of new neurons with or without inducing hair cells^7,68^ and is consistent with predictions of various cell types that require independent inducers^9,10^. Understanding how the expansion of neuronal projections in the absence of hair cells could be helpful to restore lost innervation^3,5,712,77,78^, in particular, understanding how to reinnervate the flat epithelia after long-term hearing loss, will be beneficial^79^.

### Deletion of *Sox2* and other genes affect spiral ganglion neuron development

Initially, deletion of *Sox2* was thought to eliminate all sensory neurons^80,81^; however, a transient development of vestibular neurons was recently shown^31^. A delayed loss of *Sox2* in *Isl1-cre; Sox2^f/f^* mice showed a transient development of spiral ganglion neurons with abnormal innervation to disorganized hair cells in the base but no hair cells or sensory neurons in the apex^73^. That the later-forming neurons in the apex never developed suggests that *Sox2* is essential for late neuronal development. Any similarities between different *Sox2* deletions (*Lcc, Ysb, Isl1-cre; Foxg1-cre*) remain to be investigated. *Eya1/Six1* induces *Sox2* expression to promote proneurosensory-lineage specification. Ablation of the ATPase-subunit *Brg1* or both *Eya1/Six1* results in loss of *Sox2* expression and lack of neurosensory identity, leading to abnormal apoptosis within the otic ectoderm. *Brg1* binds to two of three distal 3′ *Sox2* enhancers occupied by *Six1*, and *Brg1* binding to these regions depends on *Eya1/Six1* activity^82^. Recent work provides insight into SOX2 and NEUROD1 protein expression dynamics during neuronal differentiation. Quantification of the fluorescence intensity of nuclear proteins in immortalized multipotent otic progenitors showed expression dynamics of SOX2 and NEUROD1 from a progenitor into differentiated neurons. During neuronal differentiation, SOX2 levels decreased while NEUROD1 levels increased^69^. Evaluation of *Neurog1* was excluded because of its dual roles in both proliferation and neuronal differentiation^68^. The increase of *Neurod1* expression is in line with what is known for *Neurod1* in collaboration with *Sox2*^10,31^. Understanding the expression dynamics of crucial transcription factors helps design replacement strategies for lost sensory neurons^69^.

The deletion of *Pax2* resulted in a near absence of spiral ganglion neurons^43^, comparable to the significant loss of spiral ganglion neurons in *Isl1-cre; Sox2^f/f^* mice^73^. Many additional genes derail the development of the inner ear and its innervation^9,83–86^. For example, disorganized projections to the cochlea are shown with *Sox10* deletion in Schwann cells^87^. In addition, partial loss of hair cells reorganizes the remaining afferents and efferents^75,88,89^. These data provide a baseline of various deficits that require further examination, including the disorganized innervation in conditional deletions of *Gata3*^9,32,90^. Other genes, such as those involved in Wnt signaling, affect afferent innervation to OHCs^85^, but more work is needed to fine-tune the different effects. Finally, *Lmx1a* loss results in a delayed upregulation of *Atoh1* combined with a transformation of basal turn hair cells into a mix of cochlear and vestibular hair cells^10,13^. In summary, *Sox2* is essential for sensory neuron development^31^ in combination with other downstream neuronal inducers (*Neurog1* and *Neurod1*) known to interact with *Atoh1*^16,27^.

## Cochlear Nuclei

### *Neurod1* and *Atoh1* are expressed in the cochlear nuclei

Beyond a transient and limited expression of *Neurog1* expression in vestibular nuclei^21,91,92^, the other bHLH genes, *Atoh1* and *Neurod1*, are expressed in cochlear nuclei^18,93,94^. *Atoh1* is expressed in developing cochlear nuclei, and the dorsal cochlear nucleus specifically requires *Neurod1*^22,23^. *Atoh1* is expressed dorsally in the central nervous system and its deletion disrupted spinal cord, brainstem, and cerebellum development^95,96^. Rhombomere-specific deletion of *Atoh1* demonstrates that the cochlear nucleus forms from cells in rhombomeres 3–5^17,97^. *Atoh1* expression is negatively regulated by *Neurod1* in the cerebellum^41,98^, the cochlear hair cells and neurons^10^, and the intestine^99^ but has not yet been shown for the cochlear nucleus. An additional bHLH gene, *bHLHb5*^97^, is also necessary to properly form the dorsal cochlear nucleus. Both *bHLHb5* and another gene, *Ptf1a*, are strongly expressed in the dorsal cochlear nucleus^39,100^; however, details on central projections for losing either of those two genes have not yet been provided^94,101^. Loss of *Atoh1* or *Ptf1a* resulted in a loss of excitatory or inhibitory cochlear nuclei neurons, respectively, suggesting that both genes are important for regulating cell fate determination^38,39^. Recent molecular work on *Atoh1* and *Ptf1a* lineage contributions to cochlear nuclei development show conserved and divergent origins across species^15,102^.

**Figure 3.**
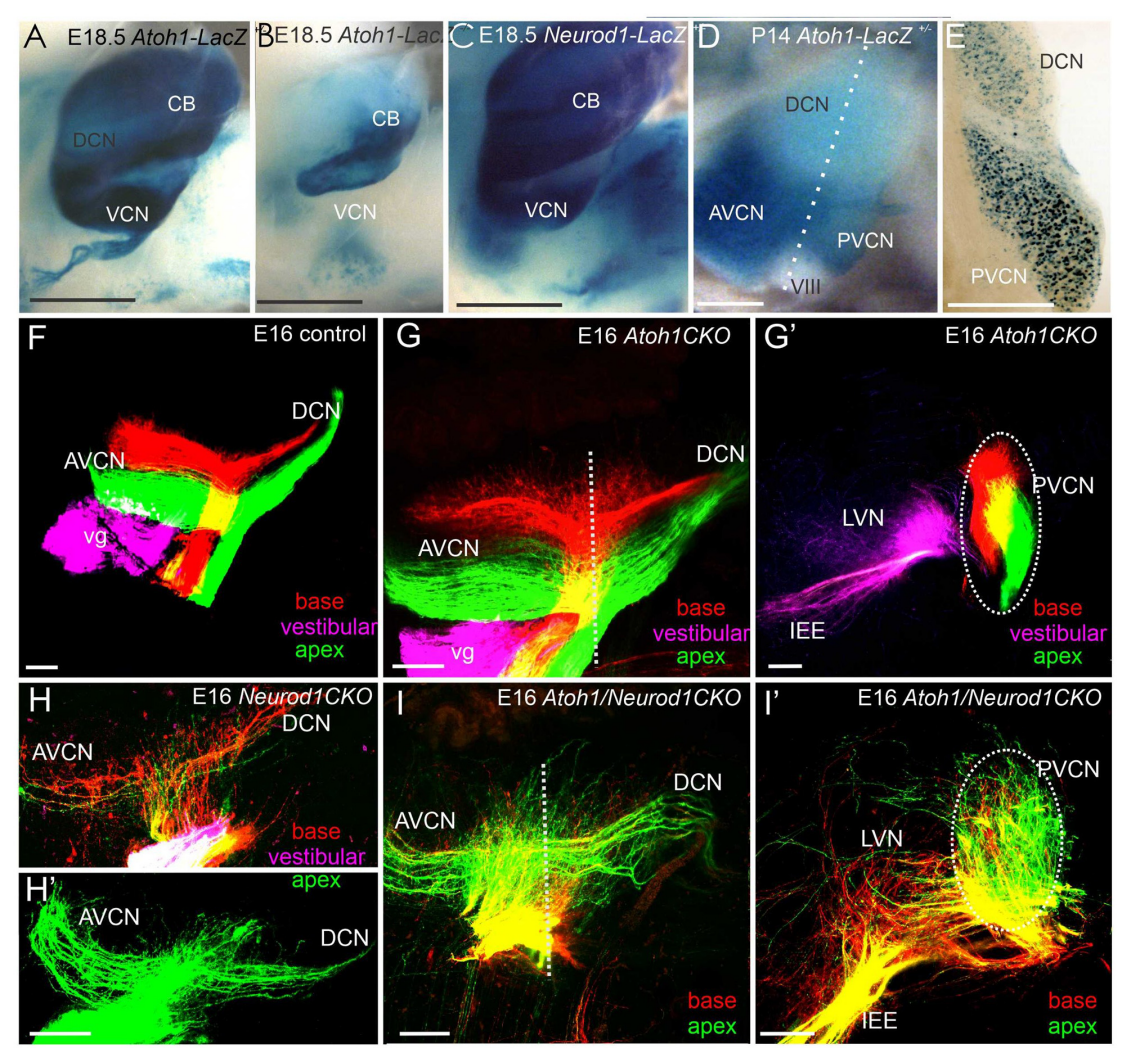
*Atoh1* is expressed in the cochlear nuclei and the cerebellum for development. Loss of *Atoh1* (*Atoh1^LacZ/LacZ^*) results in the loss of the cerebellum and cochlear nuclei (**A**, **B**). Likewise, *Neurod1* is expressed in cochlear nuclei and cerebellum (**C**). It shows later differential expression in the dorsal cochlear nucleus (DCN) (low level of *Atoh1*; (**D**)) compared with the stronger expression of *Neurod1* in the DCN (**E**), suggesting a negative feedback between *Atoh1* and *Neurod1*. The central projection of sensory neurons is nearly identical between controls (**F**) and *Atoh1* CKO mutants (**G**, **G′**). In contrast, both *Neurod1* CKO (**H**, **H′**) and *Atoh1/Neurod1* CKO mice (**I**, **I′**) show scrambled central projections. AVCN, anteroventral cochlear nucleus; CB, cerebellum; E, embryonic day; IEE, inner ear efferents; LVN, lateral vestibular nucleus; PVCN, posteroventral cochlear nucleus; VCN, ventral cochlear nucleus; vg, vestibular ganglion. This figure was adapted with permission from Fritzsch *et al*.^901^ under the terms of the Creative Commons 4.0 Attribution License (CC BY 4.0) (**A**–**E**), from Copyright Clearance Center: Springer Nature, Cell and Tissue Research, Pan *et al*.^41^, Copyright © 2009, Springer-Verlag (**D**,**E**), and from Copyright Clearance Center: Springer Nature, Molecular Biology, Filova *et al*.^27^, Copyright © 2020, Springer Nature (**F**–**I′**).

*Neurod1* deletion is shown to affect the central targeting of inner ear neurons massively. Not only are auditory neuron projections aberrant, but there is also an overlap of cochlear and vestibular projections^16^. Furthermore, the central projections are disorganized to the inferior colliculi^16^, expanding previous work on defects generated with *Hoxb2* mutants^103^. In contrast, *Atoh1* null mutants, which lack cochlear nuclei, show near-normal central projections^104^, suggesting that neither *Atoh1* nor the cochlear nuclei themselves have a notable role in afferent pathfinding centrally. The conditional deletion of *Atoh1* in the ear, but retaining *Atoh1* expression in cochlear nuclei, shows near-normal segregation of central projections^27^, expanding the critical independence of *Atoh1* in neuronal pathfinding. Not surprisingly, then, *Atoh1/Neurod1* double null mice had little additional disorganized projection of cochlear afferents beyond that of *Neurod1* alone^27^ (Figure 3). *Atoh1/Neurod1* forms a complex interaction in the cerebellum^41,98,105^, which is useful for *Neurod1* to convert astrocytes and Schwann cells into neurons^70,106,107^. Details are needed to determine whether deviations of central projections (Figure 1) would occur in older stages after cochlear nuclei are formed^30^ and dependence of cochlear nuclei on neuronal input declines^12^. Recent data suggest plastic reinnervation of cochlear nuclei^108^, but it remains unclear whether this plasticity is permanent.

These data implicate several different bHLH genes (*Atoh1*, *Neurod1*, *Ptf1a*, and *bHLHb5*) in cochlear nuclei development. The interactions of these genes in cochlear nuclei development and innervation remain to be fully characterized.

### *Sox2* and *Lmx1a/b* are expressed in cochlear nuclei

*Sox2* is essential for proneuronal regulation throughout the entire brain^109,110^ and is broadly expressed in cochlear nuclei, but its role has not been detailed by selective *Sox2* deletion in cochlear nuclei. *Lmx1a/b* double null mutants lack cochlear nuclei and choroid plexus and have a hindbrain reminiscent of a spinal cord^13^. In these mice, central projections of spiral ganglion neurons are lost, and vestibular fibers project bilaterally to the dorsal hindbrain and interdigitate with contralateral vestibular fibers^13^. The presence of these bilateral projections correlated with the expression of other genes, such as *Wnt3a* and *Tbr2*. The suggested *Wnt3a* attraction expands on previous data showing that loss of the Wnt receptor, *Fzd3*^111^, or downstream Wnt signaling component, *Prickle1*^86^, affects central projections. Recent work suggests that another gene, *Npr2*, affects central projections, showing the gain and loss of afferents to different cochlear nuclei^32,35^.

In summary, the expression of *Lmx1a/b* for the proper formation of the hindbrain is essential and the deletion of *Lmx1a/b* causes aberrational projections. In contrast to the detailed description of *Lmx1a/b* loss, there is limited information on the role of *Sox2* and other genes (*Npr2*, *Prickle1*, *Fzd3*, and *Wnt3a*) on central projections.

## Cochlear Hair Cells

### *Neurog1*, *Neurod1*, and *Atoh1* interaction in developing hair cells

**Figure 4.**
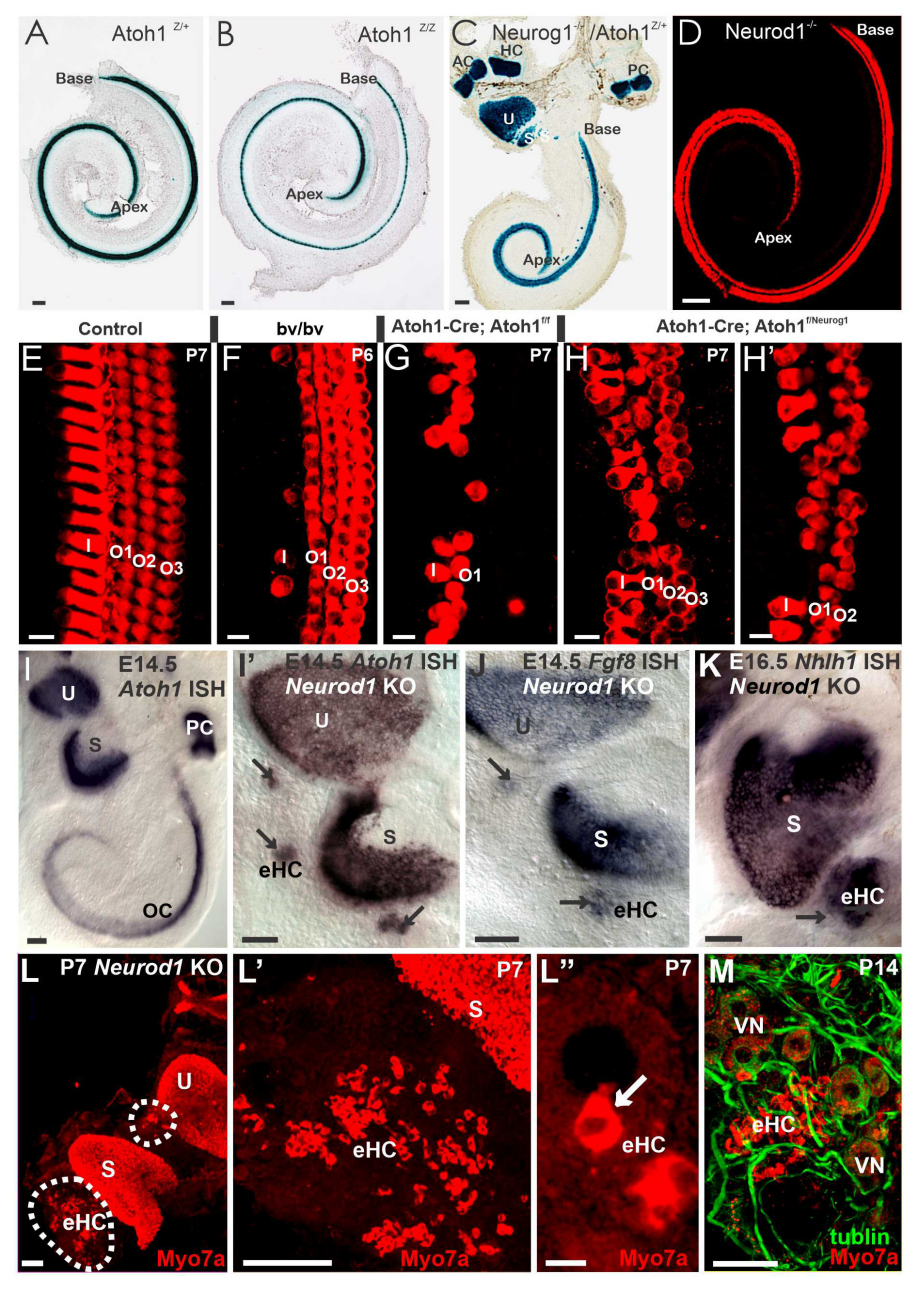
Expressed of *Atoh1* is needed for cochlear hair cells for development. Loss of *Atoh1* has a limited effect of cochlea extension (**A**, **B**) compared with the shortened cochlea in *Neurog1* (**C**) and *Neurod1* (**D**) null mice. Detailed images compare control hair cells (**E**) within Bronx waltzer (*bv/bv*) (**F**) and “self-terminating” *Atoh1^f/f^* (**G**) mice. They demonstrate near-complete loss of inner hair cells in *Atoh1^f/kiNeurog1^* mice (**H**, **H′**), demonstrating incomplete development of different sets of hair cells. Expression of Atoh1 *in situ* hybridization (ISH) depends on the normal expression pattern in control end organs (**I).** Ectopic “hair cells” after *Neurod1* deletion are shown with ISH for *Atoh1*, *Fgf8*, and *Nhlh1* (**I′**, **J**, **K**). Hair cells within vestibular epithelia (**L**) as well as ectopic hair cells (**L–L″, arrow in L”) are positive for Myo7a**. Myo7a labeling also shows ectopic hair cells innervated by tubulin-labeled vestibular neurons (VN) (**M**). AC, anterior canal crista; eHC, ectopic hair cells; HC, horizontal canal crista; P, passage; PC, posterior canal crista; S, saccule; U, utricle. Bar indicates 100 µm (**A**–**L′**, **M**) and 10 µm (**L″**). This figure was modified after Fritzsch *et al*.^58^ (**A**,**B**) and was adapted with permission from Matei *et al*.^28^ (**C**), from Jahan *et al*.^54^ under the terms of the Creative Commons Attribution License (**D**, **I**–**M**), and from Booth KT *et al*.^64^ under the terms of the Creative Commons 4.0 Attribution License (CC BY 4.0) (**E**–**H’**).

Without a doubt, the development of all hair cells depends upon *Atoh1* expression^24^. *Atoh1* expression initiates in the cochlea at the upper-middle turn around E13.5 and progresses bilaterally toward the base and apex. *Atoh1* expression shows a delayed upregulation in the apex compared with the base^24,58^, combined with very late apical hair cell differentiation at E18.5^112,113^. Interestingly, inner pillar cells were positive for *Atoh1*, suggesting that *Atoh1* expression does not always result in a hair cell fate^28,114^. In contrast to differentiation of hair cells starting near the base and progressing toward the apex, hair cells exit the cell cycle first in the apex, at E12.5, and progress toward the base^28,29,115^. Furthermore, cell exit progresses radially from IHCs to OHCs^10,116,117^, as was shown initially using green fluorescent protein (GFP) labeling^118^. Loss of *Neurog1* results in hair cells exiting the cell cycle two days earlier than controls^28^. Furthermore, there is a premature *Atoh1* upregulation in an atypical apex-to-base progression in hair cells following *Neurog1* loss^19,28^. Likewise, in *Neurod1* null mice, early upregulation of *Atoh1* from apex to base resulted in the formation of IHC-like cells in the region of OHCs, suggesting a transformation of OHCs into IHCs because of increased *Atoh1* expression^16,23^. The cellular processes driving remodeling of the prosensory domain during cochlear development indicate that combinations of cellular growth contribute to base-to-apex cochlear extension, allowing different interpretations of OHC progression^10,88,116,117,119,120^. Despite its prominent role in hair cell differentiation, *Atoh1* (Figure 4) does not seem to have a role in cochlear length determination^27^. In contrast, *Neurog1* deletion resulted in a 50% reduction in cochlear length, a reduction in the size of vestibular epithelia^28^, and ectopic hair cells in the utricle^9,121^. Likewise, loss of *Neurod1* (Figure 4) shortened the cochlea by about 50%^16,23^. *Atoh1/Neurod1* double knockout added minimally to the cochlear length reduction in *Neurod1* loss alone^27^. Although this suggests a possible interaction of bHLH genes, the reduction in length may be influenced simply by the loss of *Shh* normally generated by spiral ganglion neurons^122^, which would be absent or reduced in number in *Neurog1* or *Neurod1* null mice. The reduction of the organ of Corti is affected by several deletions of *Shh*^123^, *Gata3*^75^, *Foxg1*^45,124^, and *Lmx1a*^47,49^ in addition to *Neurog1* and *Neurod1*.

Conditional deletion of *Atoh1* using *Pax2-cre* showed that most hair cells were lost during late embryonic development; however, some undifferentiated cells express *Myo7a* in postnatal stages and are targeted by neurons. A “self-terminating” system (*Atoh1-cre; Atoh1^f/f^*), in which a transient expression of *Atoh1* results in some initial hair cell development, demonstrated progressive loss of IHCs and most OHCs shortly after birth^11^. However, some *Myo7a*-positive OHCs remained in adults in these mice. This suggests that most hair cells depend upon continued *Atoh1* expression for at least some time. Various other conditional deletions of *Atoh1* established that continued *Atoh1* expression is essential for hair cell survival and maturation^100,125^. Interestingly, generating a transgenic mouse in which *Neurog1* replaces *Atoh1* (*Atoh1^kiNeurog1/kiNeurog1^*) showed that, although *Neurog1* cannot fully rescue the *Atoh1* null hair cell loss phenotype, it does form additional patches of undifferentiated “hair cells” rather than a flat epithelium^63^. In addition, heterozygote mice expressing one copy of each gene (*Atoh1 ^kiNeurog1/+^*) showed some disorganization of hair cell distribution (Figure 2 and Figure 4) not observed in *Atoh1* heterozygotes, suggesting cross-interaction between *Atoh1* and *Neurog1*. Using an ingenious system to overexpress *Atoh1*, in which the *Atoh1* coding sequence is under the control of a tetracycline response element (TRE), generated viable ectopic “hair cells” in early postnatal mice^126^ in line with an upper induction of proliferation^127^.

Loss of *Neurod1* resulted in the formation of *Atoh1*-positive “hair cell”-like cells within intraganglionic vesicles (Figure 4) in the vestibular ganglion^54^, suggesting a potential conversion of vestibular sensory neurons into hair cells. The ectopic hair cells are forming in addition to the saccule and utricle and are positive for several genes—such as *Atoh1*, *Fgf8*, and *Nhlh1*—that generally are expressed outside the hair cells (Figure 4). This finding indicates the normal suppression of *Atoh1* by *Neurod1* in these neurons and implies that *Neurod1* might suppress hair cell fate in sensory neurons^16^. Similar *Neurod1–Atoh1* interactions were reported in the cerebellum^41,98^ and the intestine^99^ and were used to transform astrocytes to neurons^106,107^. In the absence of both *Atoh1* and *Neurod1* in double null mutants, these “ectopic hair cells” are not formed^27^, suggesting that *Neurod1* and *Atoh1* interact upregulate neurons into ectopic hair cells after the loss of *Neurod1*.

In summary, using progenitor cells for spiral ganglia and hair cell replacement seems to be a possible way forward for hearing restoration^7,68^, in addition to various other approaches^6,8,77,128^. Unfortunately, generation of new hair cells in later stages beyond the earliest stages has not yet been achieved^127^. Understanding how to generate new hair cells at later stages is needed for older animals and humans with aging-related hearing loss^1,2^. Fully understanding the various mutations and putting them into the context of different cell fates require identifying certain steps necessary to initiate specific distributions of sensory hair cells^10,113,129,130^. What remains is understanding the various interactions of *Neurog1*, *Neurod1*, and *Atoh1* for the complete formation of all hair cells.

### *Sox2* interacts with other genes for hair cell expression

*Sox2* is also essential for hair cell formation^52^, likely through the activation of *Atoh1* expression^109,110,131^. Interestingly, two independent approaches using delayed deletion of *Sox2*^53,73,131^ showed different results. In one, a delayed loss of *Sox2* using *Sox2-cre-ER* demonstrated effects in the apex only^131^. In the other study, conditional deletion of *Sox2* using *Islet1-cre* resulted in the loss of hair cells in the apex and a delayed loss in the base, showing unusual basal turn hair cells/supporting cells and inner pillar cells^73^, suggesting a role for the timing of *Sox2* expression. As expected, the timing of *Sox2* expression was later demonstrated to be essential for sensory development^81,132^. Furthermore, a complete deletion of *Sox2* in the ear using *Foxg1-cre* showed the overall cochlear reduction and no hair cell development^31^. These combined studies provide an essential role of *Sox2*, although the interaction of *Sox2* with *Atoh1* is not fully understood^6,8,68,76,77,88,117^.

Other genes are also crucial for inner ear and hair cell development. For example, *Eya1/Six1* is essential for early ear development and is needed to form the cochlea^44,50,53^ and induces *Sox2* expression, as described earlier^82^. Another gene, *Pax2*, is necessary for organ-of-Corti formation**^43^ and cooperates with *Sox2* to activate *Atoh1* expression^51^. Conditional deletion of *Gata3* using *Pax2-cre* showed deletion of many hair cells and a complete loss of all hair cells with an earlier deletion of *Gata3* using *Foxg1-cre*^42,75^. In these latter mice, levels of *Atoh1* expression were significantly reduced, and genes downstream of *Atoh1* were not detected following this early deletion of *Gata3*. Mice mutant for another gene, *Lmx1a*, showed a delayed expression of *Atoh1* followed by transforming some organ-of-Corti hair cells into differentiated vestibular hair cells^2,13,47,133^. *Foxg1* null mice show a reduced cochlear length and a disorganized apex of multiple rows of hair cells with disoriented polarities^45,46,124,134^. A somewhat similar phenotype is reported for *n-Myc* null mutants accompanied with apical cell fate changes^46,57,135–137^.

The partial deletion of some, but not other, hair cells is an exciting perspective that needs to be explored. Inactivation of *Fgfr1* in the inner ear by *Foxg1-Cre*–mediated deletion leads to an 85% reduction in the number of auditory hair cells^138^. Likewise, *Sox2* omission shows a partial loss of hair cells in the *Yellow submarine* (*Ysb*) mutation^52^. Using *Pax2-cre* to conditionally delete *Dicer*^89^ resulted in incomplete hair cell loss compared with the total hair cell loss with *Foxg1-cre* conditional deletion, comparable to the equivalent conditional deletions of *Gata3*^75,139^. Finally, Bronx-waltzer mice, which are mutant for the gene *Srrm4* (Figure 4), lose IHCs and vestibular hair cells but retain OHCs^60,61^. OHCs, meanwhile, express *Srrm3* independent of the *Srrm4* gene downstream of REST^61^.

These data show that cochlear hair cells are affected by single gene deletions and complex interactions of several genes, including compound analysis of partial deletions^10^, primarily unexplored in detail^7,72^. While *Atoh1* alone is the dominant gene^24^, interactions with other genes need to be worked out^44,77,78^.

## Summary and conclusion

Inner ear sensory neurons, cochlear nuclei, and cochlear hair cells all require bHLH genes for their proper development. *Atoh1* is essential for cochlear hair cell and cochlear nuclei development. *Neurog1* and *Neurod1* are vital for sensory neuron development and differentiation. All three genes play crucial roles in a feedback network to regulate specific cell fate appropriately and in coordination with other genes. Some of these additional genes interact with the bHLH genes in these contexts, such as *Lmx1a/b*, requiring more detailed investigation.
